# Comparison of clinical and laboratory characteristics of neuromyelitis optica spectrum disorder with or without anti-connective tissue antibodies: an 18-month cohort follow-up

**DOI:** 10.3389/fimmu.2026.1829927

**Published:** 2026-06-19

**Authors:** Shuna Shi, Yang Liu, Zhenling Fu, Junzhe Yang, Zhengyu Sun, Haiyang Luo, Limei Wang, Yuming Xu

**Affiliations:** 1 Department of Neurology, The First Affiliated Hospital of Zhengzhou University, Zhengzhou University, Zhengzhou, Henan, China; 2 Department of Neurology, Nanyang Central Hospital, Nanyang, Henan, China; 3 Henan Provincial People’s Hospital, Department of Neurology, Zhengzhou, Henan, China; 4 Tianjian Laboratory of Advanced Biomedical Sciences, School of Life Sciences, Zhengzhou University, Zhengzhou, Henan, China

**Keywords:** 18-month follow-up, anti-connective tissue antibodies, AQP4-IgG, clinical characteristics, connective tissue disease-associated autoantibodies, laboratory characteristics, neuromyelitis optica spectrum disorder

## Abstract

**Purpose:**

This study aimed to explore the significance of anti-connective tissue antibodies in the clinical diagnosis and evaluation of neuromyelitis optica spectrum disorder (NMOSD).

**Methods:**

Demographic and clinical data from 205 patients with aquaporin-4 immunoglobulin G (AQP4-IgG)-positive NMOSD were collected. Variables included sex, age, clinical symptoms/signs, connective tissue antibody status, inflammatory markers, cerebrospinal fluid (CSF) cell counts/oligoclonal band status, spinal cord lesion location/length, Expanded Disability Status Scale scores at onset and first relapse (18-month follow-up), and time to first relapse.

**Results:**

Among the 205 patients, 108 (52.7%) were positive for anti-connective tissue antibodies (CTD abs+). Compared with anti-connective tissue antibodies (CTD abs−) patients, the CTD abs+ group had higher lymphocyte counts (1.82 ± 0.14 vs. 1.73 ± 0.07, *p <* 0.01), higher monocyte-to-lymphocyte ratios [0.27 (0.2) vs. 0.24 (0.16), *p =* 0.037], and higher CSF white blood cell counts [10 (22) vs. 6 (18)/10^6^/L, *p =* 0.035]. They also showed a higher rate of oligoclonal band positivity (27.78% vs. 10.31%, *p* = 0.002), a higher proportion of patients with an increased 24-hour intrathecal synthesis rate (54.6% vs. 40.2%, *p* = 0.039), and higher CSF immunoglobulin levels [5.755 (6.37) vs. 4.15 (3.53) mg/dL, p < 0.001] at initial onset. No significant differences were observed between the CTD abs+ and CTD abs− groups in the distribution of lesions or the length of affected spinal cord vertebral segments at initial onset. However, patients in the CTD abs+ group had higher Expanded Disability Status Scale scores at first relapse and a higher annualized relapse rate over the 18-month follow-up period(*p* < 0.05for both).

**Conclusions:**

In summary, among AQP4-IgG-positive patients with NMOSD, CTD abs+ was associated with higher lymphocyte counts, a higher MLR, higher CSF white blood cell counts, higher CSF immunoglobulin levels, a higher 24-hour intrathecal IgG synthesis rate, and higher OCB positivity. CTD abs positivity may therefore indicate a more severe inflammatory profile; however, it does not appear to predict earlier relapse, but does appear to predict a higher annualized relapse rate.

## Introduction

Neuromyelitis optica spectrum disorder (NMOSD) is an autoimmune disease of the central nervous system (CNS) that can lead to a range of clinical syndromes involving the optic nerve and spinal cord (1, 2). It is primarily caused by pathogenic autoantibodies that specifically target aquaporin-4 (AQP4) proteins located on the foot processes of astrocytes (3). AQP4-IgG autoantibodies exhibit high specificity and affinity for AQP4 antigenic sites, triggering a complement cascade and exacerbating inflammatory responses, which leads to disruption of the blood–brain barrier (BBB), astrocyte damage or dysfunction, subsequent demyelination, and ultimately the onset and progression of NMOSD (4). Several studies have reported that NMOSD may coexist with systemic autoimmune diseases, with these conditions occurring either before or after the onset NMOSD (5, 6). The presence of comorbid autoimmune diseases may increase the annual relapse rate in patients with NMOSD (7, 8).

Some autoantibodies can be detected many years before the clinical manifestations of autoimmune diseases appear, referred to as the preclinical phase of autoimmune disease (9). Previous studies have reported an increased prevalence of autoimmune antibodies in the general population (10), which may result from the dysregulation of immune response mechanisms and altered immune tolerance (11). Patients with NMOSD may exhibit multiple non-organ-specific autoantibodies, such as connective tissue and thyroid antibodies. Connective tissue antibodies include antinuclear antibodies (ANA), anti-double-stranded DNA antibodies (anti-dsDNA), anti-Sm antibodies, anti-SSA/Ro and anti-SSB/La antibodies, and anti-Scl-70 antibodies, among others. The reported connective tissue antibody (CTD abs) positivity rates vary among patients with NMOSD (ANA, 27.3–51.5%; anti-SSA, 10.5–36.5%; anti-SSB, 0–9.3%; anti-dsDNA, 0–7.8%) (12). However, studies examining whether CTD abs positivity (CTD abs+) increases disease activity or recurrence in NMOSD are limited, and their findings remain inconsistent (13, 14).

The potential pathological mechanisms underlying CTD abs−mediated effects in NMOSD include: (1) direct axonal injury, where anti−SSA antibodies binding to cell−surface SSA antigens may directly induce axonal degeneration and, in some cases, loss of myelinated fibers (15); (2) CTD abs promoting the upregulation of proinflammatory cytokines, such as interleukin−6 and tumor necrosis factor−α, thereby intensifying intrathecal inflammation (16); and (3) CTD abs contributing to BBB disruption (7).

It therefore remains unclear whether patients with NMOSD with or without CTD abs+ exhibit different clinical and/or laboratory characteristics. To further investigate the clinical significance of CTD abs+ in the diagnosis and evaluation of NMOSD, the present study analyzed the relationship between CTD abs status and clinical and laboratory characteristics in 205 patients with NMOSD and AQP4-IgG positivity.

## Materials and methods

### Participants and data collection

**Figure 1:**
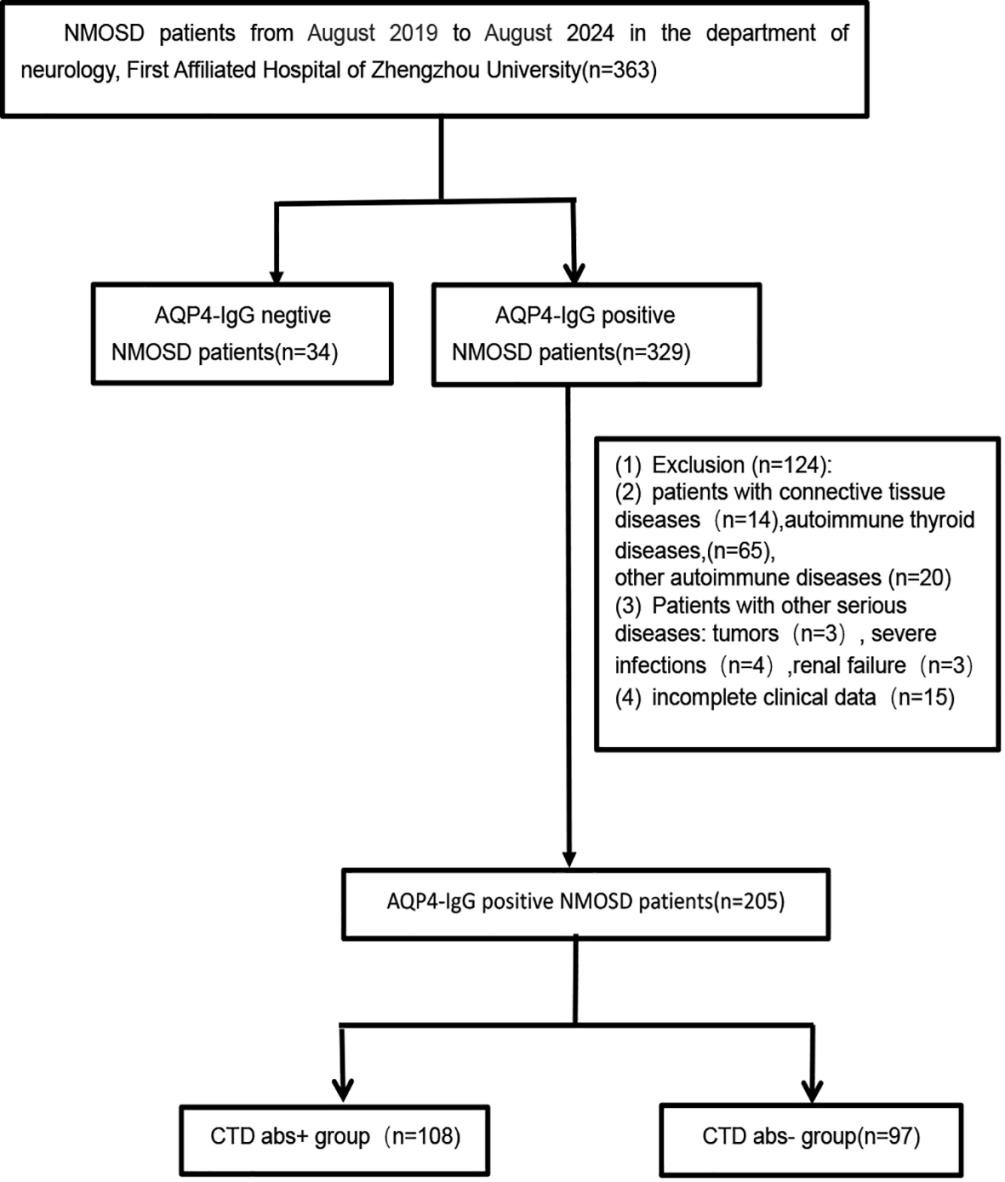
Flowchart of patient selection for the study. AQP4-IgG, aquaporin-4 immunoglobulin G; NMOSD, neuromyelitis optica spectrum disorder; CTD abs+, positive for anti-connective tissue antibodies.

In this study, 363 patients diagnosed with NMOSD and admitted to the Department of Neurology at the First Affiliated Hospital of Zhengzhou University between August 2019 and August 2024 diagnosed with NMOSD were initially screened. Among them, 205 patients with AQP4-IgG positivity were included in the final analysis (**Figure 1**). This study was approved by the Ethics Committee of the First Affiliated Hospital of Zhengzhou University (Approval number: 2021-KY-0822-002).

All included patients (1) fulfilled the 2015 International Panel for NMO diagnostic criteria (17), (2) experienced their first episode of disease, (3) were ≥18 years old, and (4) tested positive for AQP4-IgG based on a cell-based assay, with an antibody titer ≥1:10 considered positive. Clinical data were collected for all patients, including sex, age, clinical symptoms and signs, CTD abs status, inflammatory indicators, cerebrospinal fluid (CSF) cell counts and oligoclonal band (OCB) status, the location and length of affected spinal cord vertebral segments, Expanded Disability Status Scale (EDSS) scores at initial onset and at the first relapse during the 18-month follow-up, and the annualized relapse rate over the 18-month follow-up period, with the EDSS scores independently assessed by two neurologists.

The exclusion criteria were: (1) the presence of other CNS demyelinating diseases, including multiple sclerosis, myelin oligodendrocyte glycoprotein antibody−associated disease, or acute disseminated encephalomyelitis; (2) a diagnosis of any connective tissue disease (CTD) (e.g., systemic lupus erythematosus, Sjögren’s syndrome, rheumatoid arthritis, vasculitis) or other autoimmune disease (e.g., autoimmune thyroid disease, myasthenia gravis) prior to NMOSD onset. Patients with a prior formal CTD diagnosis were excluded (all such patients were CTD antibody positive); only those who were CTD antibody positive without a prior clinical CTD diagnosis were included in the CTD abs+ group. (3) the use of corticosteroids (e.g., prednisone ≥re mg/day or equivalent for >2 weeks) within 30 days prior to study enrollment, or the use of immunosuppressive therapy (including but not limited to rituximab, tocilizumab, ofatumumab, eculizumab, mycophenolate mofetil, sulfasalazine, methotrexate, cyclophosphamide, or tacrolimus) within 6 months prior to enrollment; (4) the presence of severe comorbid conditions, including heart failure (New York Heart Association class III or IV), liver failure (Child–Pugh class C), end−stage renal disease, active infection within 30 days, malignancy, or hematological diseases; (5) pregnancy or breastfeeding at the time of enrollment; (6) loss to follow−up before 18 months (these patients were included in the analyses only for the duration they were followed, but were excluded from the primary 18−month outcome analysis); and (7) incomplete clinical data for key variables (e.g., missing AQP4−IgG or CTD abs results or missing baseline EDSS score).

Fresh paired serum and CSF samples were obtained within 30 days after disease onset and before the administration of corticosteroids and immunosuppressive therapy. Samples were stored at 2–8 °C and analyzed within 15 days. All patients received corticosteroid therapy and disease-modifying therapy (DMT). Both the duration of DMT and the follow-up period exceeded 18 months. Clinical relapse was defined as the occurrence of new neurological deficits more than 1 month after the initial episode (18).

### Complete blood count

Upon admission, fasting blood samples were collected to measure neutrophil, monocyte, lymphocyte, and platelet counts. Based on these parameters, the following inflammatory indices were calculated: neutrophil-to-lymphocyte ratio (NLR), monocyte-to-lymphocyte ratio (MLR), platelet-to-lymphocyte ratio (PLR), systemic inflammation response index (SIRI), calculated as monocytes × NLR, and systemic immune-inflammation index (SII), calculated as platelets × NLR (19).

### Detection of connective tissue antibodies

CTD abs were detected using the following methods. ANA, anti-dsDNA, and anti-neutrophil cytoplasmic antibodies were detected using indirect immunofluorescence assays (20). Anti-SSA, anti-SSB, anti-Ro52, anti-nRNP/Sm, anti-histone, and anti-ribosomal P antibodies were detected using chemiluminescence immunoassays. The results were interpreted according to hospital-specific reference values (20).

### Evaluation of the blood–CSF barrier and intrathecal IgG synthesis

An elevated albumin quotient (QAlb), defined as greater than the age-related quotient ([4 + age/15] × 10^−3^), was considered to indicate disruption of the blood–CSF barrier (21). An IgG index >0.7 (22) and an IgG synthesis rate >10 (23) were considered indicative of intrathecal IgG synthesis.

### OCB detection

OCBs were detected using isoelectric focusing on agarose gels followed by immunoblotting, which is the recommended method for OCB analysis. Paired undiluted CSF and serum samples (24) were analyzed simultaneously. Two independent investigators interpreted the electrophoresis results according to the following classification: type I, no bands in serum or CSF; type II, more than two bands in CSF with no corresponding serum bands; type III, CSF-restricted bands in addition to bands present in both CSF and serum; type IV, identical bands in both serum and CSF; and type V, monoclonal bands with regular spacing in both serum and CSF (25). Types II and III were considered positive for CSF OCBs (OCB+), whereas the remaining types were considered negative (OCB−).

### Statistical analysis

Statistical analyses were performed using SPSS version 27.0 (IBM Corp., Armonk, NY, USA). Sample size was estimated using G*Power software (26)based on an expected difference in OCB positivity rates (31.3% in patients with CTD abs+ vs. 8.7% in patients with CTD abs−) (27). A two-group comparison of independent proportions (two-sided z-test) was performed. With α = 0.05 and power = 0.95, 66 patients per group were required. Accounting for a 10% dropout rate, the target sample size was set at 147patients (approximately 74 per group). The final enrollment (N = 205) exceeded this requirement. The median time to first relapse was estimated using the Kaplan–Meier method, and differences between groups were assessed using the log-rank test. Annualized relapse rates were calculated as the total number of relapses divided by the total follow-up time (in years) during the 18-month study period. Negative binomial regression was used to compare annualized relapse rates between groups, with the logarithm of follow-up time included as an offset. The results are presented as rate ratios with 95% confidence intervals (CIs). The normality of continuous variables was assessed using the Shapiro–Wilk test. Normally distributed data are presented as the mean ± standard deviation, whereas non-normally distributed data are expressed as the median with the interquartile range (IQR) in parentheses, i.e., median (IQR). Categorical variables were compared between groups using Pearson’s Χ^2^ test, Fisher’s exact test, or the Cochran–Mantel–Haenszel test, as appropriate. Continuous variables were compared using an independent-samples t-test or the Mann–Whitney U-test, depending on data distribution. A *p*-value < 0.05 was considered statistically significant.

## Results

### Demographic data and clinical manifestations at enrollment

**Figure 2:**
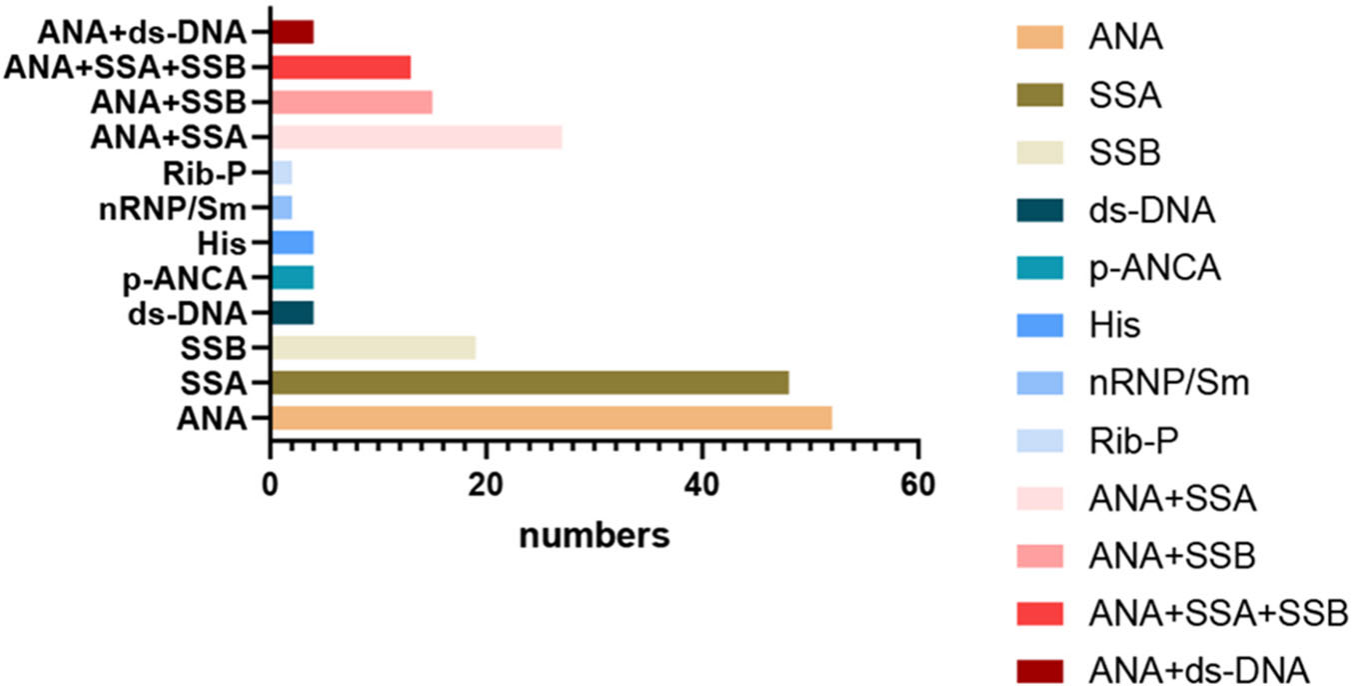
Distribution of connective tissue antibodies among patients with connective tissue antibody positivity. The proportions were as follows: ANA, 52/108 (48.2%); anti-SSA, 48/108 (44.4%); anti-SSB, 19/108 (17.6%); ds-DNA, 4/108 (3.7%); p-ANCA, 4/108 (3.7%); His, 4/108 (3.7%); nRNP/Sm, 2/108 (1.9%); Rib-P, 2/108 (1.9%); ANA and SSA, 27/108 (25.0%); ANA and SSB, 5/108 (13.9%); ANA, SSA, and SSB, 13/108 (12.0%). ANA, antinuclear antibody; SSA, syndrome-related antigen A; SSB, anti-SSB antibodies; ds-DNA, anti-double-stranded DNA antibodies; p-ANCA, perinuclear type anti neutrophil nuclear antibody; His, anti-histone antibodies; nRNP/Sm, anti-nRNP/Sm antibodies; Rib-P, anti-ribonucleic acid antibodies.

A total of 205 patients with NMOSD were enrolled in this study, including 44 men and 161 women (male-to-female ratio = 1:3.7; **Figure 1**). The CTD abs+ group comprised 108 patients, including 11 men and 97 women (male-to-female ratio = 1:8.8), with a mean age of 46.42 ± 15.15 years. The CTD abs− group comprised 97 patients, consisting of 33 men and 64 women (male-to-female ratio = 1:1.9), with a mean age of 50.25 ± 15.30 years. The proportion of men was significantly lower in the CTD abs+ group than in the CTD abs− group (11/108 vs. 33/97, *p <* 0.001). However, there were no significant differences in age distribution between the two groups (*p* = 0.073). Among the 108 patients in the CTD abs+ group, 52 (48.1%) were positive for ANA, 48 (44.4%) for anti-SSA antibodies, and 19 (17.59%) for anti-SSB antibodies. Additionally, 27 patients (25%) were positive for both ANA and SSA, 15 (13.9%) for ANA and SSB, and 13 (12%) for ANA, SSA, and SSB (**Figure 2**). There was no significant difference in baseline EDSS scores between the CTD abs+ and CTD abs− groups [4.0(2.5) vs. 3.5 (2.5), *p* = 0.127].

### Comparison of CNS lesions on magnetic resonance imaging between CTD abs+ and CTD abs− groups at enrollment

Baseline magnetic resonance imaging evaluation showed that optic nerve lesions were present in 30 of 108 (27.8%) patients in the CTD abs+ group and in 23 out of 97 (23.7%) in the CTD abs− group. Spinal cord abnormalities were the most common imaging feature, occurring in 85 of 108 (78.7%) patients in the CTD abs+ group and in 83 of 97 (85.6%) in the CTD abs− group. Long-segment spinal cord lesions were observed in 67 of 108 (62%) patients in the CTD abs+ group and in 50 of 97 (51.5%) in the CTD abs− group. Simultaneous optic nerve and spinal cord lesions were detected in 14 of 108 (13%) patients in the CTD abs+ group and in 7 of 97 (7.2%) in the CTD abs− group. Brain or brainstem involvement was identified in 47 of 108 (43.5%) patients in the CTD abs+ group and in 48 of 97 (49.5%) in the CTD abs− group. No statistically significant differences were observed between the two groups in terms of lesion distribution patterns (all *p* > 0.05) (**Table 1**).

**Table 1:** Demographic and clinical characteristics of AQP4-IgG-positive NMOSD patients stratified by CTD abs status.

Variable	CTD abs+	CTD abs-	Z value or χ² value	*P* value
Male, n (%)	11/108,10.2%	33/97,34.0%	17.223	***< 0.001***
Onset age(year), mean ± SD	46.42 ± 15.15	50.25 ± 15.30		0.073
Time from onset to testing(days), median (IQR)	16.5 (11)	17 (9)	-0.223	0.823
EDSS at enrollment, median (IQR)	4.0 (2.5)	3.5 (2.5)	-1.527	0.127
EDSS at the first recurrence during the 18-month follow-up, median (IQR)	4.0 (1.0)	2.0 (1.0)	-6.084	***< 0.001***
EDSS at 18-month follow-up, median (IQR)	3.0 (2.5)	2.0 (1.0)	-3.923	***< 0.001***
DMTs, n (%)			6.691	0.153
AZA	6 (5.6%)	6 (6.2%)		
MMF	65 (60.2%)	70 (72.2%)		
RTX	6 (5.6%)	7 (7.2%)		
Other Efficient DMTs	26 (24.1%)	13 (13.4%)		
Others	5 (4.6%)	1 (1.0%)		
Imaging lesions evidenced by MRI at enrollment, n (%)				
Involving the optic nerve	30/108,27.8%	23/97,23.7%	0.441	0.507
Involving the spinal cord	85/108,78.7%	83/97,85.6%	1.627	0.202
Involving the optic nerve and spinal cord	14/108,13.0%	7/97,7.2%	1.835	0.175
Involving long-segment spinal cord injury(≥4)	67/108,62.0%	50/97,51.5%	2.296	0.130
Involving the brain and brainstem	47/108,43.5%	48/97,49.5%	0.731	0.392

EDSS, Expanded Disability Status Scale; CSF, cerebrospinal fluid; CTD, connective tissue disease; IQR, interquartile range; DMTs, disease-modifying therapies; AZA, azathioprine; MMF, mycophenolate mofetil; RTX, rituximab. Other effective DMTs include Satralizumab, ofatumumab, and Inebilizumab. “Other” therapies include tacrolimus, cyclophosphamide, and hydroxychloroquine. *P* values are presented in *italics*, and values <0.05 are highlighted in *bold italics*. Data are presented as mean ± SD or median (IQR).

To evaluate the potential influence of medication on laboratory indicators, differences in treatment regimens between the two groups were also analyzed. No significant differences in treatment were observed between CTD abs+ and CTD abs− patients (*p* = 0.153) (**Table 1**).

### Comparison of CSF parameters between CTD abs+ and CTD abs− patients

Regarding CSF biochemical findings, the CTD abs+ group had higher CSF white blood cell counts than the CTD abs− group [10 (22) vs. 6 (18)/10^6^/L, *p* = 0.035]. Detailed CSF cell differentiation analysis revealed that 125 of the 205 patients (61%) showed lymphocyte-predominant CSF pleocytosis, including 67 of 108 (62%) patients in the CTD abs+ group and 58 of 97 (59.8%) in the CTD abs− group (*p* = 0.742). The predominant finding in both groups was a reduced proportion of CSF mononuclear cells; however, no statistically significant difference was observed between the groups (85/108, 78.7% vs. 67/97, 69.1%; *p* = 0.116). There was also no significant difference in CSF protein levels between the groups (471.77 ± 311.57 mg/L vs. 417.68 ± 232.62 mg/L; *p* = 0.132).

In the CTD abs+ group, 59 of 108 patients (54.6%) had an elevated 24-hour IgG synthesis rate, compared with 39 of 97 patients (40.2%) in the CTD abs− group. The proportion of patients with elevated IgG synthesis rate was significantly higher in the CTD abs+ group (*p* = 0.039) (**Table 2**). Furthermore, the CTD abs+ group had higher CSF immunoglobulin levels than the CTD abs− group [5.755 (6.37) mg/dL vs. 4.15 (3.53) mg/dL, *p* < 0.001]. (**Table 2**). The OCB positivity rate was also significantly higher in the CTD abs+ group than in the CTD abs− group (30/108, 27.78% vs. 10/97, 10.31%; *p* = 0.002).

**Table 2:** Comparison of immune-inflammatory indicators at enrollment stratified by CTD abs status.

Variable	CTD abs+	CTD abs-	Z value	*P* value
Immune-inflammation indicators				
WCC (×10^9^/L), mean ± SD	7.45 ± 0.35	7.13 ± 0.32		0.728
NEU (×10^9^/L), mean ± SD	5.19 ± 0.32	5.07 ± 0.34		0.986
LYM (×10^9^/L), mean ± SD	1.82 ± 0.14	1.73 ± 0.07		***< 0.010***
MON (×10^9^/L), median (IQR)	0.41 (0.28)	0.4 (0.25)	-0.8	0.424
PLT (×10^9^/L), mean ± SD	238.34 ± 69.27	247.15 ± 64.12		0.349
PLR, median (IQR)	148.19 (103.51)	154.6 (79.94)	-0.08	0.939
NLR, median (IQR)	2.62 (3.04)	2.36 (2.77)	-0.696	0.487
MLR, median (IQR)	0.27 (0.2)	0.24 (0.16)	-2.085	***0.037***
SII (×10^9^/L), median (IQR)	606.19 (673.43)	579.6 (639.26)	-0.093	0.926
SIRI (×10^9^/L), median (IQR)	1.075 (1.75)	0.88 (1.04)	-1.306	0.191
CSF profile				
CSF WCC (×10^6^/L), median (IQR)	10 (22)	6 (18)	-2.11	***0.035***
CSF LYM increased, n (%)	67/108,62.0%	58/97,59.8%	0.108	0.742
CSF MON decreased, n (%)	85/108,78.7%	67/97,69.1%	2.473	0.116
IgG index, median (IQR)	0.735 (0.4)	0.69 (0.29)	-0.869	0.385
24h IgG synthesis rate increased, n (%)	59/108,52.8%	39/97,40.2%	4.261	***0.039***
OCB+, n (%)	30/108,27.78%	10/97,10.31%	9.929	***0.002***
Qalb, median (IQR)	5.84 (3.47)	5.35 (3.21)	-0.982	0.326
Proportion of Qalb elevation, n (%)	74/108,65.52%	55/97,56.70%	3.059	0.08
QIg, median (IQR)	4.37 (4.72)	3.88 (2.77)	-1.155	0.248
CSF Ig (mg/dL), median (IQR)	5.755 (6.37)	4.15 (3.53)	-3.576	***< 0.001***
Serum Ig (mg/dL), median (IQR)	1247.75 (622.05)	1102.5 (323.6)	-2.968	*0.003*
CSF albumin (mg/dL), median (IQR)	24.585 (19.47)	23.55 (14.08)	-1.388	0.165
SER albumin (mg/dL), median (IQR)	4197.5 (564.25)	4236 (491.5)	-1.053	0.292

WCC, white blood cell count; LYM, lymphocytes; MON, monocytes; NEU, neutrophils; PLT, platelets; NLR, neutrophil-to-lymphocyte ratio; MLR, monocyte-to-lymphocyte ratio; PLR, platelet-to-lymphocyte ratio; SIRI, systemic inflammation response index (monocytes × NLR); SII, systemic immune inflammation index (platelets × NLR); OCB+, oligoclonal band positivity; CSF, cerebrospinal fluid; QAIb, albumin quotient index; SER, serum; Ig, immunoglobulin; QIg, immunoglobulin quotient index; OCB, oligoclonal band. *P* values are presented in *italics*, and values <0.05 are highlighted in *bold italics*. Data are presented as mean ± SD or median (IQR).

### Comparison of peripheral blood inflammatory indicators

Compared with the CTD abs− group, the CTD abs+ group had significantly higher lymphocyte counts (*p* < 0.01). The median MLR was 0.27 (0.2) in the CTD abs+ group and 0.24 (0.16) in the CTD abs− group, with a statistically significant difference between groups (*p* = 0.037). However, no significant differences were observed between the groups in the total white blood cell count, monocyte count, platelet count, PLR, NLR, SII, or SIRI (all *p >* 0.05).

### Comparison of clinical manifestations between CTD abs+ and CTD abs− groups

At enrollment, myelitis was present in 85 of 108 (78.7%) patients in the CTD abs+ group and in 83 of 97 (85.6%) in the CTD abs− group. Optic neuritis (ON) occurred in 32 of 108 patients (29.6%) in the CTD abs+ group and in 27 of 97 (27.8%) in the CTD abs− group. No statistically significant differences were observed between the groups in the incidence of myelitis or ON (*p >* 0.05). Area postrema syndrome occurred in 19 patients (17.6%) in the CTD abs+ group and in 6 (6.2%) in the CTD abs− group (*p =* 0.013). During the 18-month follow-up, relapses occurred in 42 patients in the CTD abs+ group. These relapses manifested as myelitis in 31 patients (73.8%), ON in 9 patients (21.4%), and area postrema syndrome in 2 patients (4.8%). In the CTD abs− group, 28 patients experienced relapses, presenting as myelitis in 19 patients (67.9%), ON in 4 patients (14.3%), and area postrema syndrome in 2 patients (7.1%). No statistically significant differences were observed between the two groups in relapse manifestations (all *p* > 0.05) (**Table 3**).

**Table 3:** Clinical syndromes of AQP4-IgG-positive patients with NMOSD stratified by the presence or absence of CTD antibodies.

Characteristic	CTD abs+ (n=108)	CTD abs- (n=97)	χ² value	*P* value	Cramer’s V
Onset syndrome					
Optic neuritis	32/108, 29.6%	27/97, 27.8%	0.080	0.777	0.020
Myelitis	85/108, 78.7%	83/97, 85.6%	1.627	0.202	0.089
Area postrema syndrome	19/108, 17.6%	6/97, 6.2%	6.210	***0.013***	0.174
Relapses during the 18-month follow-up					
Recurrence rate	42/108, 38.9%	28/97, 28.9%	2.283	0.131	0.106
Optic nerve	9/42, 21.4%	4/28, 14.3%	0.567	0.452	0.090
Spinal cord	31/42, 73.8%	19/28, 67.9%	0.252	0.589	0.065
Area postrema syndrome	2/42, 4.8%	2/28, 7.1%		> 0.999	0.050

*P* values are presented in *italics*, and values <0.05 are highlighted in *bold italics*.

There was no significant difference in baseline EDSS scores between the groups [4 (2.5) vs. 3.5 (2.5), *p =* 0.127]. However, at the 18-month follow-up, the CTD abs+ group had significantly higher EDSS scores at the time of first relapse than the CTD abs− group[4 (1) vs. 2 (1), *p <* 0.001].

**Figure 3:**
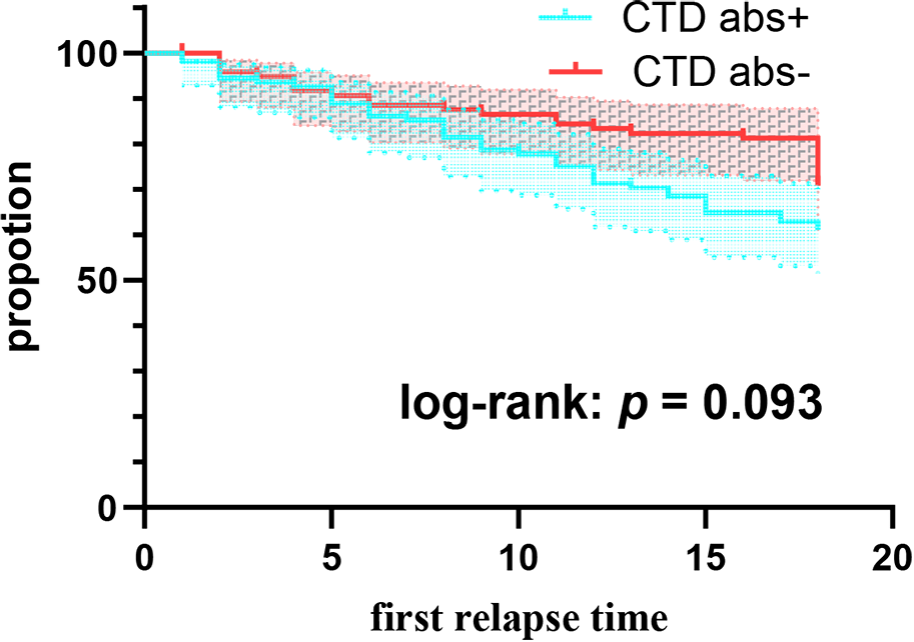
Kaplan–Meier survival analysis of time to first relapse in patients with NMOSD according to connective tissue antibody (CTD abs) status. The median time to first relapse was not significantly different between the CTD abs+ and CTD abs− groups (11 vs. 11 months; log-rank test, *p =* 0.093).

Kaplan–Meier survival analysis showed that the median time to first relapse in the CTD abs+ group was not earlier than that in the CTD abs− group (11 vs. 11 months, log-rank test *p =* 0.093) (**Figure 3**). The relapse rate within 18 months was 38.9% in the CTD abs+ group and 28.9% in the CTD abs− group (*p =* 0.131). The annualized relapse rate during the 18-month follow-up period was 0.340 (95% CI: 0.267–0.432) in the CTD abs+ group and 0.227 (95% CI: 0.178–0.289) in the CTD abs− group. Negative binomial regression revealed that CTD abs positivity was associated with a significantly higher relapse rate, with a rate ratio of 1.50 (95% CI: 1.10–2.05, p = 0.011).

## Discussion

ANA and other non-organ-specific autoantibodies are frequently detected in patients with NMOSD who lack clinical evidence of systemic autoimmune diseases (28). ANA and anti-SSA antibodies are among the most commonly detected autoantibodies in NMOSD and are closely associated with AQP4 antibody seropositivity (12, 29). In the present study, among patients with NMOSD and AQP4-IgG and CTD abs+, the prevalence of ANA positivity was 52/108 (48.15%), and that of anti-SSA antibodies was 48/108 (44.44%). Consistent with previous reports, ANA showed the highest positivity rate.

Previous studies have reported female−to−male ratios of approximately 2–3:1 for antinuclear antibody (ANA) positivity and 3:1 for SSA antibody positivity in the healthy population, suggesting that women may be a high−risk cohort for CTD abs+ (30–32). Epidemiological data have indicated a female-to-male ratio of approximately 4:1 in global NMOSD populations (33). In our cohort of 108 patients with NMOSD with CTD abs+, the sex distribution was markedly skewed, with 89.8% being female (female-to-male ratio of 8.8:1), which exceeds both the ratios reported in healthy populations and those in general NMOSD cohorts. While direct statistical comparisons with external populations are not possible due to the absence of a concurrent control group, this observation raises the possibility that CTD abs+ may influence the sex distribution of NMOSD, further supporting a role for sex differences in NMOSD pathogenesis (34).

Previous studies have indicated that patients with CTD abs+ exhibit a higher proportion of OCB positivity and increased 24-hour intrathecal IgG synthesis compared with CTD abs− patients. Notably, an elevated 24-hour intrathecal IgG synthesis rate has been identified as an independent risk factor for increased disease activity in AQP4-IgG-positive patients with NMOSD (35, 36). In the present study, CTD abs+ patients also exhibited higher rates of OCB positivity and elevated 24-hour intrathecal IgG synthesis compared with CTD abs− patients. Additionally, EDSS scores at the time of the first relapse were higher in the CTD abs+ group. These findings are consistent with previous reports and suggest that the presence of connective tissue disease antibodies should be considered when evaluating disease activity in AQP4-IgG-positive patients with NMOSD.

Yang et al. (27) reported predominantly monocyte-rich CSF in patients with NMOSD. However, their study did not further differentiate CSF cell types into lymphocytes, monocytes, neutrophils, eosinophils, or basophils. In contrast, the present study further classified CSF cell types and found that, regardless of CTD abs status, CSF was predominantly lymphocyte-rich, mainly with increased lymphocytes and decreased monocytes. However, the proportions of elevated lymphocytes and reduced monocytes were not significantly higher in the CTD abs+ group than in the CTD abs− group. Future studies with larger sample sizes are warranted to further clarify the potential influence of CTD abs on CSF cellular composition.

Anti-AQP4 autoantibodies bind to the AQP4 antigen on astrocytic endfeet. This interaction triggers the classical pathway of the complement cascade, inflicting primary damage to astrocytes via the assembly of the membrane attack complex and antibody-dependent cellular cytotoxicity (37, 38). Glial fibrillary acidic protein is a marker for astrocytic injury in patients with NMOSD with autoantibody positivity (39). Additionally, serum interleukin-6 (40, 41), glial fibrillary acidic protein (42), and neurofilament light chain (43) levels are linked to BBB permeability in NMOSD and may influence the extent of disability in affected patients. Previous studies have reported that patients with connective tissue diseases may present with CNS symptoms and abnormal CSF findings (27, 44), suggesting that CTD abs may influence CSF parameters. In the present study, CSF white blood cell counts, immunoglobulin levels, and OCB positivity rates were higher in the CTD abs+ group than in the CTD abs− group. This may be related to disruption of the BBB, infiltration of immune cells, and increased cytokine production. Together, these observations suggest that CTD abs+ patients with NMOSD may exhibit more pronounced inflammatory activity. Moreover, CTD abs may promote intrathecal IgG synthesis in NMOSD and influence CSF cellular and immunological characteristics (32).

Previous studies have demonstrated that neutrophils and inflammatory monocytes can exacerbate inflammation within the central nervous system (CNS) (45–48). In addition, B cells, neutrophils, and eosinophils infiltrate the CNS across the BBB and contribute to the development of NMOSD lesions (49, 50). Both neutrophils and lymphocytes have been identified in AQP4-IgG-induced NMOSD animal models as well as in active lesions in patients with early NMOSD (45, 51). However, in the present study, no statistically significant differences were observed in neutrophil or monocyte counts between the CTD abs+ and CTD abs− groups at disease onset, a finding that may be attributable to the relatively small sample size. Notably, significant differences were found in lymphocyte counts and the monocyte-to-lymphocyte ratio (MLR). These findings suggest that, among AQP4-IgG-positive patients with NMOSD, the presence of connective tissue antibodies may be associated with higher peripheral lymphocyte counts and an elevated MLR. The MLR has been reported to be a useful predictor of NMOSD recurrence (52). Additionally, previous studies have demonstrated that EDSS scores, lesion burden, and relapse frequency during the acute phase of NMOSD are positively correlated with inflammatory markers such as the MLR, NLR, PLR, SII, and SIRI (53). Future studies with expanded cohorts are warranted to elucidate whether CTD antibody status influences other peripheral inflammatory markers in NMOSD, and whether disease-modifying therapies for NMOSD alter lymphocyte counts, the MLR, or the serological status of CTD antibodies.

Compared with the CTD abs+ group, the CTD abs− group showed no differences in the time to first relapse, relapse rate, or lesion distribution at relapse. However, EDSS scores at the time of first relapse were higher in the CTD abs+ group, suggesting that the presence of connective tissue antibodies may be associated with greater disease severity and may require more aggressive treatment. NMOSD is characterized by relatively high relapse and disability rates, and acute relapses often lead to the accumulation of neurological disability (1). Previous studies have suggested that anti-SSA antibody positivity may be a risk factor for NMOSD relapse (54, 55) and may be associated with worse EDSS outcomes. ANAs seem to be associated with more severe disease activity in NMOSD (56). In the present study, EDSS scores at first relapse within 18 months were higher in the CTD abs+ group than in the CTD abs− group. Previous studies have shown that patients with NMOSD with Sjögren’s disease have higher relapse rates than those without Sjögren’s disease (57). Moreover, patients with NMOSD with coexisting connective tissue diseases have been reported to experience earlier and more frequent relapses (58), suggesting that CTD abs positivity may influence NMOSD recurrence even in the absence of clinically diagnosed connective tissue disease. Notably, CTD abs+ patients in our cohort showed significantly higher EDSS scores at recurrence, annual recurrence rates, and OCB positivity rates. These differences may be linked to the pathological mechanisms driven by CTD abs in NMOSD, including direct axonal injury from anti-SSA antibody binding (15), promotion of proinflammatory cytokine upregulation (16), as well as disruption of the BBB (7), which is a known feature of NMOSD that facilitates AQP4−IgG access to CNS astrocytes (59).

Appropriate DMTs can reduce relapse frequency and slow disease progression. Therefore, when comparing relapse timing and disability severity, treatment regimens should be considered potential confounding factors. In the present study, no significant differences in treatment were observed between groups based on CTD abs status. However, during the 18-month follow-up, a significant difference in disease severity at first relapse was observed between the two groups, suggesting that CTD abs positivity may predict more severe disease in NMOSD.

This study had several limitations. First, only AQP4-IgG-positive patients with NMOSD were included owing to the greater heterogeneity of AQP4-negative NMOSD. Second, comparing CTD antibody prevalence between NMOSD and other CNS disorders (e.g., multiple sclerosis, MOGAD) was beyond our scope. Moreover, no published study has directly compared CTD antibody positivity between NMOSD and truly non-inflammatory CNS conditions (e.g., migraine without aura, tension-type headache) using standardized methods. Most available data come from diseases with secondary neuroinflammation, including acute stroke (23.0% ANA positivity) (60), Parkinson’s disease (12% ANA positivity) (61), multiple system atrophy (8% ANA positivity) (61), dementia with Lewy bodies (18% ANA positivity) (61), and brain tumors (85% for antinucleosomal antibodies) (26)—conditions that are unsuitable as “non-inflammatory” comparators. The closest benchmark is healthy controls, with an ANA positivity rate of 17% (61) in one study, considerably lower than the 48.15% observed in our cohort. However, healthy controls cannot account for whether chronic neurological morbidity itself influences CTDabs prevalence. Future large-scale, multicenter investigations incorporating both inflammatory and non-inflammatory CNS control groups are needed to clarify the diagnostic and pathogenic value of these antibodies. Third, the late approval of satralizumab and inebilizumab in China limited the use of these highly effective disease-modifying therapies (other than rituximab), which may have influenced the observed relapse rate and interval, although no significant treatment differences were identified between groups. Fourth, the absence of post-treatment CTD abs measurements precluded our ability to determine whether the observed inflammatory profile and relapse risk were driven by persistent CTD abs positivity or by other factors. It remains possible that CTD abs status may change over time or in response to immunotherapy, which could have implications for disease monitoring and treatment stratification. Future studies incorporating serial CTD abs assessments are needed to address this question. Fifth, this was a single-center retrospective study with a relatively small sample size, and all enrolled cases were of Asian ethnicity. Consequently, the generalizability of our findings is limited, and larger multicenter, multi-ethnic prospective studies are required to validate these results.

In summary, among AQP4-IgG-positive patients with NMOSD, CTD abs+ was associated with higher lymphocyte counts, a higher MLR, higher CSF white blood cell counts, higher CSF immunoglobulin levels, a higher 24-hour intrathecal IgG synthesis rate, and higher OCB positivity. CTD abs positivity may therefore indicate a more severe inflammatory profile; however, it does not appear to predict earlier relapse, but does appear to predict a higher annualized relapse rate.

## Data Availability

The original contributions presented in the study are included in the article/supplementary material. Further inquiries can be directed to the corresponding author.
